# A study on the Perception of General Practitioners of a Locality in Kolkata Regarding RNTCP and DOTS

**DOI:** 10.4103/0970-0218.66883

**Published:** 2010-04

**Authors:** Aparajita Dasgupta, Amitabha Chattopadhyay

**Affiliations:** Department of Community Medicine, All India Institute of Hygiene and Public Health, 110-C.R. Avenue, Kolkata, West Bengal, India

## Introduction

The Revised National Tuberculosis Control Program (RNTCP), based on the internationally recommended directly observed treatment, short course (DOTS) strategy, was initially implemented mainly through the network of healthcare facilities under the public health departments of the state governments. Studies have shown that the private sector is the first point of contact for more than half of the TB patients.

In most countries with a significant burden of TB, DOTS implementation is limited largely to public sector services under the tuberculosis program. In reality, however, many patients with symptoms of TB, including the very poor, do seek and receive care from a wide variety of healthcare providers outside the network of the tuberculosis program and these providers do not always coordinate with the national program or apply to DOTS.

In order to address this apparent weakness in global TB control, efforts have been under way within and outside the World Health Organization’s (WHO’s) Public-Private Mix (PPM) initiative. The obvious goal of developing PPM is to promote access to quality TB care worldwide. The RNTCP has recognized the Indian Medical Association (IMA) as a major partner in expanding PPM DOTS service in this country.

Therefore, the contribution of the general practitioners for the control and management of tuberculosis must not be overlooked or neglected. To bring their method of treatment under the umbrella of RNTCP is the need of the hour. For this, a baseline data, to assess their knowledge and practice regarding RNTCP/DOTS is urgently required. With this background a study has been conducted to find out the perception of the general practitioners, of a locality in Kolkata, regarding RNTCP and DOTS.

## Materials and Method

This was a cross-sectional observational study done in Behala and Alipore area in Kolkata for a period of six months (March 2008 to August 2008). The study population included general practitioners enlisted in IMA, Behala, and IMA, Alipore, some general practitioners not enlisted in IMA, and few RMPs (rural medical practitioners). The total sample size was 233.

A pre-designed, pre-tested, semi-structured, self-administered questionnaire was used, and informed consent from the participants was taken beforehand. The questionnaire had ten knowledge and five practice-related questions. Knowledge and practice were separately scored. [Three or less=poor knowledge; Four to seven=average knowledge; >seven=good knowledge (maximum score=ten, minimum score =0)][Two or less=poor practice; Three or more=good practice (maximum score=five, minimum score =0)]. Statistical analysis was done by using Epi info 3.4.3 version.

## Results

In Table 1, the following was observed. Correct response regarding the full form of RNTCP and DOTS was given by 63.9 and 27% of the practitioners, respectively. Again, the most common symptom of TB and number of treatment categories under RNTCP were rightly answered by 21 and 39% of the practitioners, respectively. Correct responses regarding anti-TB drug contraindicated in pregnancy and whether DOTS was applicable for children was given by about 60% of the practitioners, while first line anti-TB drugs and frequency of drug administration in DOTS was correctly stated by less than 30% of the practitioners. Correct method of sputum collection was known to about 44% of the participants. Only 9.9% of the physicians registered and forwarded information to the District Tuberculosis Center (DTC). Two or three sputum examinations as the first examination for detecting TB was practiced by only about 35%, while health education regarding preventive practices was given to the patients by only 22% of the physicians. Supervised treatment of patients and advice regarding isoniazid prophylaxis to household contact aged less than 5 years was given by less than 20% of the practitioners.

**Table 1:** Correct knowledge and practice of private practitioners regarding RNTCP and DOTS (n=233)

Information sought -* (in bracket, correct responses given)	No. of correct responses (%)
Full form of RNTCP - (Revised National Tuberculosis Control Program)	149 (63.9)
Full form of DOTS - (directly observed treatment, short course)	63 (27)
Most common symptom of TB - (cough for three weeks or more)	49 (21)
No. of treatment categories under RNTCP - (3)	91 (39)
Anti TB drug contraindicated in pregnancy - (streptomycin)	139 (59.6)
Anti TB drugs that should be stopped in jaundice - (all)	108 (46.4)
Correct method of sputum collection - (spot-morning-spot)	102 (43.8)
Frequency of drug administration in DOTS - (thrice weekly)	54 (23.2)
Whether DOTS is applicable to children - (yes)	140 (60)
First line anti TB drugs - (i/r/p/e/s)#	68 (29.2)
Register and send information to DTC - (yes)	23 (9.9)
First exam for detecting TB - (2 or 3 sputum exams)	82 (35.2)
Supervised treatment - (yes)	34 (14.6)
Advice given to under five household contacts - (INH prophylaxis)	45 (19.3)
Health education given regarding preventive practices -	52 (22.3)

# (i-isoniazid, r- rifampicin, p-pyrazinamide, e-ethambutol, s-streptomycin).

* (Q1 -10 → information related to knowledge, Q 11-15→information related to practice).

Good knowledge and proper practice was present in 24 and 19.7% of the practitioners, respectively. About 68% of the practitioners sent TB patients to private laboratories, while 31% of the practitioners sent their patients to the government sector for diagnosis. About 95% of the general practitioners agreed to the fact that involvement of general practitioners was important in combating TB in India, and were willing to be active partners in the implementation of RNTCP/DOTS.

## Discussion

India has the highest global burden of TB and the largest private medical sector.[1] For most patients, including those with symptoms of TB, the first part of call is often a neighborhood private doctor. The private healthcare providers are a heterogeneous, largely unregulated group, and include those qualified in the Western and indigenous systems of medicine as well as non-qualified practitioners.

In the present study only 9.9% of the private practitioners sent information to the District Tuberculosis Center. In a study from Pakistan[2] this rate was about 6%. About 35% of the practitioners stated that two or three sputum examinations were their first priority for diagnosis, and about 39% knew about the three treatment categories in tuberculosis management, which was however, more than that in a study in Chandigarh,[3] where it was only 3%. In a study among interns in Delhi,[4] 48.4% of the participants correctly knew that under RNTCP, treatment is given on thrice-a-week intermittent basis, while the correct response rate in this study was 23.2% only. About 29.2% of the study subjects correctly named all the five first-line anti-tubercular drugs of RNTCP, which was similar to a study in Delhi (26.5%). Supervised treatment for patients was given by less than 20% of the practitioners. Overall a good knowledge and proper practice was present only in 24 and 19.7% of the private practitioners, respectively. However, it was felt that a bigger sample size, from a wider geographical area, would have been more representative for the assessment of the actual perception of private medical practitioners.

## Conclusion

For a national program to broaden its reach and have maximal impact, the involvement of private practitioners assumes great importance. It is one of the big challenges policy makers face in India, to ensure the participation of these practitioners, which is intimately linked with the success of the program. Anti-tubercular drugs can be provided through the private practitioners after imparting the necessary training to them. Although the study revealed gaps and weakness in the private doctors with regard to knowledge and practice of RNTCP and DOTS, it was observed that a majority of the private practitioners wanted to be a part of RNTCP. This willingness could be utilized properly for building a public-private partnership, for control of tuberculosis in India.
